# Prevalence of anxiety and depression among Palestinian university students: a cross-sectional study during COVID-19 pandemic

**DOI:** 10.1186/s43045-022-00238-5

**Published:** 2022-09-19

**Authors:** Mustafa Ghanim, Maha Rabayaa, Sameeha Atout, Nihad Al-Othman, Malik Alqub

**Affiliations:** Faculty of Medicine and Health Sciences, An-Najah National University, West Bank, PO. Box 7, Nablus, Palestine

**Keywords:** Anxiety, Depression, COVID-19, GAD-7, Mental health, Students

## Abstract

**Background:**

Anxiety and depression are more common among university students than in the general population. It is reported internationally that the COVID-19 pandemic increased the prevalence of anxiety and depression among students. This study aimed to measure the prevalence of anxiety and depression among university students in Palestine and to explore the association between anxiety and depression during the COVID-19 pandemic. A cross-sectional questionnaire-based descriptive study was carried out on university students. The Generalized Anxiety Disorder Scale (GAD-7) and the Center for Epidemiological Studies Depression (CES-D) scale were used to evaluate anxiety and depression, respectively. Chi-square was used to evaluate the risk factors associated with the levels of anxiety and depression. The association between anxiety and depression was evaluated using Pearson correlation.

**Results:**

A total of 1049 students were enrolled in the study. The prevalence of depression among them was 55.8%, 26.4%, and 9.8% for severe, moderate, and mild depression, respectively. Depression symptoms were significantly variable based on students’ field and year of study, sleeping period, smoking, and food source. The prevalence of anxiety was 16.1%, 30.7%, and 36.1% for severe, moderate, and mild anxiety, respectively. Anxiety symptoms were significantly variable based on students’ gender, year of study, sleeping period, smoking, and place of residence. A strong positive correlation was observed between the CES-D and GAD-7 scores (*R* = 0.707).

**Conclusion:**

Palestinian university students suffer from elevated levels of anxiety and depression during the COVID-19 pandemic. This necessitates the need for strategies by stakeholders to take immediate and appropriate interventions.

## Background

The coronavirus disease 2019 (COVID-19) pandemic pushed all the governments around the world to take radical preventive measures to limit the rapid spread of the disease [1]. In light of the absence of effective anti-viral treatment or sufficient vaccination at the beginning of the pandemic, the strategy adopted by the governments was concentrated on measures that limited social contact. These measures included—but were not limited to isolating confirmed affected cases and tracing quarantine of their close contacts. Avoiding crowds in closed and/or poorly ventilated areas was also adopted for the public leading to the lockdown of schools and universities and the transition to distance learning [1]. These preventive measures led to a negative psychological impact on the public which was exaggerated by the misinformation about COVID-19 updates that were disseminated through social media. Indeed, increased levels of anxiety and depression were noted in several international cohorts including the public and particularly among students [2].

It is anticipated that university students are more prone to psychological effects than the general population. That is because the university period is critical, in which students are subjected to many changes and challenges that put them at a higher risk of developing anxiety and depression.

In Palestine, the lockdown measures were adopted between March 2020 and August 2021 through which the universities were nearly closed. There are few studies on the psychological impact of the pandemic on the general Palestinian population [3, 4] and among school students [5]. In both cases, the levels of depression and anxiety were reported to be high in most of the studied populations. However, no systemic studies measured the psychological impact of the COVID-19 pandemic on university students. Determining the prevalence of anxiety and depression among university students will assist in developing and taking the right actions by the stakeholders. This is vital because untreated depression and to lesser extent anxiety among university students could lead to thoughts of self-harm or suicide.

### Objectives

The current study investigated the prevalence of anxiety and depression among Palestinian university students using the Center for Epidemiological Studies Depression (CES-D) scale for depression and the Generalized Anxiety Disorders (GAD-7) scale for anxiety. It also aimed to determine the risk factors associated with anxiety and depression and the correlation between them.

## Methods

### Ethical consideration

This study received ethical approval from the Institutional Review Board at An-Najah National University. Following the principles of the Declaration of Helsinki (DOH), the students agreed to participate voluntarily. The study maintained that the participants’ anonymity and confidentiality would be maintained.

### Participants

The minimal sample size was calculated to be 385 participants (95% confidence and a margin of error of 5%) using Jekel’s equation [6]. The current descriptive cross-sectional study was conducted from September to December 2021 during the COVID-19 pandemic on 1049 Palestinian university students who were selected randomly and recruited by the official university websites. The study included bachelor’s students through the whole Palestinian universities across the West Bank in the majors of human medicine, health sciences, natural sciences, engineering, or human sciences who have been accepted to be enrolled. University students at palestinian universities who signed the consent form and answer all the questions were enrolled in the study. Students who had not accepted to participate or had not completed all questions of the questionnaire were excluded.

### Study questionnaire

The study is a web-based electronic questionnaire. The questionnaire of the study was constructed based on an extensive review of related topics and involved three sections: the first was the participant demographic data which included the sex, field of study, year of study, place of residence, source of food, and smoking. The second section measured the anxiety level using the Generalized Anxiety Disorders (GAD-7) scale [7]. The last section measured the depression level using the Center for Epidemiological Studies Depression (CES-D) scale CES-D test [8].

### Assessment of anxiety symptoms

Anxiety was evaluated using the GAD-7 assessment questionnaire, which is used as a screening tool to measure the severity of generalized anxiety disorder [9]. It measures the frequency of seven anxiety symptoms that occurred in the last 2 weeks. A four-point Likert scale was used to calculate the total GAD-7 score; the scores of 0, 1, 2, and 3 were assigned to the response categories of “not at all,” “several days,” “more than half of the days,” and “nearly every day,” respectively. The sum of the responses ranged from 0 to 21, where an increase in the score is associated with more anxiety symptoms. The anxiety severity was determined by cut values: 0–4 no anxiety, 5–9 mild, 10–14 moderate, and 15–21 severe anxiety. A score of 10 or greater on the GAD-7 represents a reasonable cut point for identifying cases of GAD. GAD-7 internal consistency is excellent and a Cronbach *α* value of 0.92 was reported [10].

### Assessment of depression symptoms

Depression was evaluated using the CES-D scale, which consists of twenty items measuring how the participants felt or behaved in the previous week. A four-point Likert scale was used to calculate the total CES-D score. The scores of 0, 1, 2, and 3 were assigned to the response categories of “rarely or none of the time,” “some or a little of the time,” “occasionally or a moderate amount of time,” and “most or all of the time,” respectively, for most items which are negative in content. In contrast, items 4, 8, 12, and 16 are positive in content and the scores were reversed for them according to the questionnaire instructions where the scores of 3, 2, 1, and 0 were assigned to the response categories of “rarely or none of the time”, “some or a little of the time,” “occasionally or a moderate amount of time,” and “most or all of the time,” respectively. The sum of the scores ranged from 0 to 60, where the higher CES-D scale is associated with more depressive symptoms. The depression severity was determined by cut values: 0–9 no depression, 10–15 mild, 16–24 moderate, and 25–6 severe depression. CES-D cutoff score of 16 is adequate for the diagnosis of clinical depression. The internal consistency of the CES-D scale using coefficient alpha was estimated to be 0.85 for the general population [8, 11].

### Statistical analysis

All statistical analyses were conducted using IBM Statistical Package for the Social Sciences Statistics (SPSS) for Windows, version 21 (IBM Corp., Armonk, N.Y., USA). Descriptive analyses were used for sociodemographic characteristics. The chi-square test was used to determine the differences in the anxiety and depression severities based on the variability of the sample demographic characteristics. Bivariate Pearson correlation was used to determine the correlation between anxiety and depression scores. A *p* value of < 0.05 was considered statistically significant.

## Results

### Participant characteristics

A total of 1049 students were enrolled: 61.6% were females and 38.4% were males. The mean age was 19.8 years (SD = 1.325). They were undergraduates from different majors at the Palestinian universities and were categorized into four categories: human medicine (32.7%), health sciences (22.3%), scientific non-medical majors (19.1%), and literary majors (25.9%). With regard to lifestyle habits, 61.3% reported the average normal sleeping period for adults (6–8 h/day), 70.9% were not smokers, and 52.5% were dependent on both homemade food and restaurants. Most of the students were either living in cities or villages. A detailed description of the participants’ characteristics is shown in Table 1.

**Table 1:** Descriptive statistics of the sample characteristics (*n* = 1049)

Variable		Frequency (*n*)	Percentage (%)
1. Gender	Male	403	38.4
	Female	646	61.6
2. Field of study	Human medicine	343	32.7
	Health sciences other than human medicine	234	22.3
	Scientific non-medical majors	200	19.1
	Literary majors	272	25.9
3. Study year	First year	196	18.7
	Second year	315	30
	Third year	214	20.4
	Fourth year	191	18.2
	Fifth year	119	11.3
	Sixth year	14	1.3
4. Sleeping duration/day	Less than 6 h	266	25.4
	6 to 8 h	643	61.3
	More than 8 h	140	13.3
5. Smoking	No	744	70.9
	Yes	305	29.1
6. Source of food	Homemade food	307	29.3
	Restaurants	191	18.2
	Homemade food and restaurants	551	52.5
7. Place of residence	Village	511	48.7
	City	473	45.1
	Camp	65	6.2

### Prevalence of depression and its association with the sample characteristics

The majority of students were suffering from anxiety and depression with the predominance of depression. The overall prevalence of depression symptoms based on the CES-D test was 55.8%, 26.4%, and 9.8% for severe, moderate, and mild depression, respectively. Depression severities are shown in Table 2.

**Table 2:** Prevalence of anxiety and depression based on severity

Scale	Severity	Frequency (*n*)	Percentage (%)
CES-D scale	No depression (1–9)	84	8
	Mild (10–15)	103	9.8
	Moderate (16–24)	277	26.4
	Severe (> 24)	585	55.8
GAD-7 scale	No anxiety (< 5)	179	17.1
	Mild (5–9)	379	36.1
	Moderate (10–14)	322	30.7
	Severe (> 14)	169	16.1

The depression severity was significantly variable based on the field and the year of study, duration of daily sleeping, smoking, and the source of food (*p* value < 0.05). However, the place of residence was not a significant variable.

Based on the field of study, severe depression was more prevalent among human medicine students (60.3% of them had severe depression) followed by those who were studying scientific non-medical specialties (57.5%), literary majors (56.3%), and the least percentage of severe depression was among health sciences students (47%).

The percentage of severe depression was increasing progressively among students with increasing the number of years at the university. They were 42.3%, 53.7%, 60.3%, 61.8%, 63%, and 78.6% for the first-year students to the sixth year, respectively.

Students who were sleeping normal average hours per day (6 to 8 h) had a lesser percentage of severe depression (49.8%). However, students who were not sleeping sufficient duration (< 6 h) and who were sleeping for a longer duration (> 8 h) showed more severe depressive symptoms (63.2% and 69.3%, respectively).

More severe depressive symptoms were observed among smokers (66.2%) compared with non-smokers (51.5%). Students who were completely dependent on restaurant food had more severe depression (73.8%) compared with those who were completely or partially dependent on homemade food. Results are fully demonstrated in Table 3.

**Table 3:** The association between depression severity and sample characteristics (*n* = 1049)

Variable		Depression severity based on CES-D score				*χ*^2^	*p* value
		No depression (1–9)	Mild (10–15)	Moderate (16–24)	Severe (> 24)		
Gender	Male	32 (7.9%)	29 (7.2%)	101 (25.1%)	241 (59.8%)	6.946	0.074
	Female	52 (8.0%)	74 (11.5%)	176 (27.2%)	344 (53.3%)		
Field of study	Human medicine	27 (7.9%)	29 (8.5%)	80 (23.3%)	207 (60.3%)	19.973	**0.018**
	Health sciences	28 (12.0%)	33 (14.1%)	63 (26.9%)	110 (47.0%)		
	Scientific non-health majors	13 (6.5%)	14 (7.0%)	58 (29.0%)	115 (57.5%)		
	Literary majors	16 (5.9%)	27 (9.9%)	76 (27.9%)	153 (56.3%)		
Study year	First year	29 (14.8%)	33 (16.8%)	51 (26.0%)	83 (42.3%)	55.604	**0.000**
	Second year	21 (6.7%)	30 (9.5%)	95 (30.2%)	169 (53.7%)		
	Third year	12 (5.6%)	21 (9.8%)	52 (24.3%)	129 (60.3%)		
	Fourth year	15 (7.9%)	19 (9.9%)	39 (20.4%)	118 (61.8%)		
	Fifth year	6 (5.0%)	0 (0.0%)	38 (32.0%)	75 (63.0%)		
	Sixth year	1 (7.2%)	0 (0.0%)	2 (14.3%)	11 (78.6%)		
Sleeping duration/day	Less than 6 h	16 (6.0%)	24 (9.0%)	58 (21.8%)	168 (63.2%)	26.747	**0.000**
	6 to 8 h	59 (9.2%)	68 (10.6%)	196 (30.5%)	320 (49.8%)		
	More than 8 h	9 (6.4%)	11 (7.9%)	23 (16.4%)	97 (69.3%)		
Smoking	No	70 (9.4%)	91 (12.2%)	200 (26.9%)	383 (51.5%)	30.097	**0.000**
	Yes	14 (4.6%)	12 (3.9%)	77 (25.2%)	202 (66.2%)		
Source of food	Homemade food	28 (9.1%)	35 (11.4%)	80 (26.1%)	164 (53.4%)	45.29	**0.000**
	Restaurants	3 (1.6%)	2 (1.0%)	45 (23.6%)	141 (73.8%)		
	Homemade food and restaurants	53 (9.6%)	66 (12.0%)	152 (27.6%)	280 (50.8%)		
Place of residence	Village	35 (6.8%)	53 (10.4%)	121 (23.7%)	302 (59.1%)	8.997	0.174
	City	45 (9.5%)	46 (9.7%)	134 (28.3%)	248 (52.4%)		
	Camp	4 (6.2%)	4 (6.2%)	22 (33.8%)	35 (53.8%)		

### Prevalence of anxiety and its association with the sample characteristics

When it comes to anxiety, 36.1% of students had mild anxiety, 30.7% had moderate anxiety, and only 16.1% had severe anxiety. Anxiety severities are shown in Table 2.

Anxiety severity varied significantly between students based on their gender, year of study, sleeping duration, smoking, source of food, and place of residence. However, the field of study was not a significant factor.

Female students experienced more severe anxiety (18.3%) compared with male students (12.7%). Severe anxiety was more frequent among sixth-year students (35.7%) followed by third-year students (22%), and it was less frequent among first-year students (10.7%).

Students who were sleeping the normal average hours daily (6 to 8 h) had a lesser percentage of severe anxiety (12.9%). However, students who were not having sufficient sleeping duration (< 6 h) (22.9%) and who were sleeping for a longer duration (> 8 h) (17.9%) showed more severe anxiety symptoms.

Smoker students had a lower percentage of severe anxiety (14.4%) compared with non-smokers (16.8%). For the source of food, the severe anxiety was the lowest among students who are completely dependent on homemade food. Unlike depression, anxiety severity was significantly variable based on the place of residence. Those who were living in villages and camps had mild to moderate anxiety while those who were living in cities had either severe anxiety or no anxiety at all. The results are fully shown in Table 4.

**Table 4:** The association between anxiety severity and sample characteristics (*n* = 1049)

Variable		Anxiety severity based on GAD-7 score				*χ*^2^	*p* value
		No anxiety (< 5)	Mild (5–9)	Moderate (10–14)	Severe (> 14)		
Gender	Male	75 (18.6%)	140 (34.7%)	137 (34.0%)	51 (12.7%)	8.438	0.038
	Female	104 (16.1%)	239 (37.0%)	185 (28.6%)	118 (18.3%)		
Field of study	Human medicine	47 (13.7%)	116 (33.8%)	114 (33.2%)	66 (19.2%)	12.236	0.200
	Health sciences	49 (20.9%)	89 (38.0%)	65 (27.8%)	31 (13.2%)		
	Scientific non-health majors	39 (19.5%)	69 (34.5%)	58 (29.0%)	34 (17.0%)		
	Literary majors	44 (16.2%)	105 (38.6%)	85 (31.3%)	38 (14.0%)		
Study year	First year	47 (24.0%)	76 (38.8%)	52 (26.5%)	21 (10.7%)	30.928	0.009
	Second year	48 (15.2%)	122 (38.7%)	95 (30.2%)	50 (15.9%)		
	Third year	40 (18.7%)	68 (31.8%)	59 (27.6%)	47 (22.0%)		
	Fourth year	23 (12.0%)	73 (38.2%)	65 (34.0%)	30 (15.7%)		
	Fifth year	19 (16.0%)	37 (31.1%)	47 (39.5%)	16 (13.4%)		
	Sixth year	2 (14.3%)	3 (21.4%)	4 (28.6%)	5 (35.7%)		
Sleeping duration/day	Less than 6 h	38 (14.3%)	97 (36.5%)	70 (26.3%)	61 (22.9%)	19.968	0.003
	6 to 8 h	121 (18.8%)	239 (37.2%)	200 (31.1%)	83 (12.9%)		
	More than 8 h	20 (14.3%)	43 (30.7%)	52 (37.1%)	25 (17.9%)		
Smoking	No	129 (17.3%)	285 (38.3%)	205 (27.6%)	125 (16.8%)	12.457	0.006
	Yes	50 (16.4%)	94 (30.8%)	117 (38.4%)	44 (14.4%)		
Source of food	Homemade food	64 (20.8%)	106 (34.5%)	97 (31.6%)	40 (13.0%)	17.083	0.009
	Restaurants	29 (15.2%)	60 (31.4%)	75 (39.3%)	27 (14.1%)		
	Homemade food and restaurants	86 (15.6%)	213 (38.7%)	150 (27.2%)	102 (18.5%)		
Place of residence	Village	65 (12.7%)	197 (38.6%)	170 (33.3%)	79 (15.5%)	18.521	0.005
	City	101 (21.4%)	157 (33.2%)	131 (27.7%)	84 (17.8%)		
	Camp	13 (20.0%)	25 (38.5%)	21 (32.3%)	6 (9.2%)		

### The correlation between anxiety and depression

**Fig. 1:**
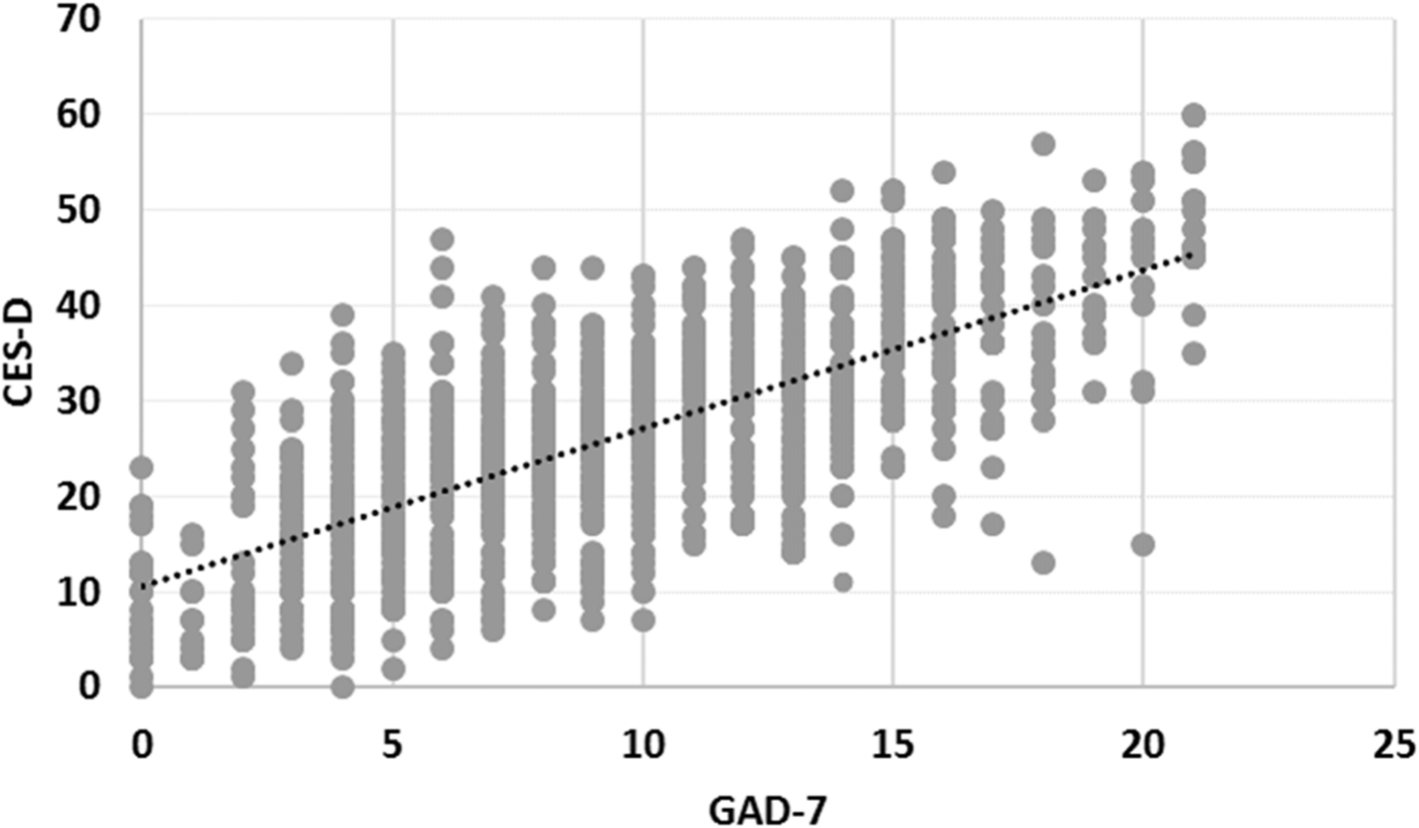
The correlation between anxiety and depression (Pearson correlation = 0.707**, Sig(two-tailed) = 0.000)

The association between anxiety and depression was evaluated using Pearson correlation, and there was a strong positive correlation between CES-D and GAD-7 scores (*R* = 0.707, *p* value < 0.01). The result is shown in Fig. 1.

### Multiple logistic regression analysis of the associations between anxiety and influencing factors

Multiple logistic regression using the anxiety score as a dependent variable, the cut value of 10 was used to evaluate the impact of variables on having a GAD-7 score of more than 10. The analysis shown in Table 5 revealed that sleeping more than 8 h has a significant protective effect against anxiety (OR = 0.675, 95% CI = 0.463–0.984). Being a smoker is also significantly protective against anxiety (OR = 0.688, 95% CI = 0.500–0.947).

**Table 5:** Multiple logistic regression analysis of the associations between anxiety and influencing factors

Variable (reference)	*B*	*p* value	OR	Lower (95% confidence)	Upper (95% confidence)
Field of study (human medicine)					
Health sciences	.287	.129	1.332	.920	1.929
Scientific non-health majors	− .025	.909	.976	.639	1.489
Literary majors	− .079	.682	.924	.633	1.349
Gender (male)					
Female	− .235	.115	.791	.591	1.059
Study year (first year)					
Second year	− .972	.102	.378	.118	1.213
Third year	− .634	.277	.531	.169	1.663
Fourth year	− .488	.407	.614	.194	1.944
Fifth year	− .520	.377	.595	.188	1.884
Sixth year	− .632	.294	.532	.163	1.730
Sleeping duration (less than 6 h)					
6 to 8 h	− .166	.446	.847	.552	1.298
More than 8 h	− .393	**.041**	.675	.463	.984
Smoking (no)					
Yes	− .374	**.022**	.688	.500	.947
Source of food (homemade food)					
Restaurants	− .124	.405	.883	.659	1.183
Homemade food and restaurants	.127	.518	1.136	.772	1.672
Place of residence (village)					
City	.477	.085	1.611	.937	2.771
Camp	.281	.309	1.325	.770	2.278

### Multiple logistic regression analysis of the associations between depression and influencing factors

In multiple logistic regression using the depression score as a dependent variable, the cut value of 16 was used to evaluate the impact of variables on having a CES-D score of more than 16 which is the value that increases the risk of clinical depression. The analysis shown in Table 6 revealed that being a smoker is significantly protective against clinical depression (OR = 0.471, 95% CI = 0.285–0.777). Using homemade food and restaurant was found to increase the risk of depression compared with using homemade food only (OR = 6.767, 95% CI = 2.612–17.529). The results are presented in Table 6.

**Table 6:** Multiple logistic regression analysis of the associations between depression and influencing factors

Variable (reference)	*B*	*p* value	OR	Lower (95% confidence)	Upper (95% confidence)
Field of study (human medicine)					
Health sciences	− .386	.154	.679	.399	1.156
Scientific non-health majors	− .379	.188	.684	.389	1.204
Literary majors	− .363	.193	.696	.403	1.201
Gender (male)					
Female	− .232	.244	.793	.537	1.171
Study year (first year)					
Second year	− 1.460	.176	.232	.028	1.928
Third year	− .655	.543	.520	.063	4.290
Fourth year	− .815	.452	.442	.053	3.698
Fifth year	− 1.083	.317	.339	.041	2.825
Sixth year	− .066	.954	.936	.099	8.890
Sleeping duration (less than 6 h)					
6 to 8 h	.186	.550	1.205	.654	2.221
More than 8 h	− .244	.375	.784	.457	1.343
Smoking (no)					
Yes	− .754	**0.003**	.471	.285	.777
Source of food (homemade food)					
Restaurants	− .083	.653	.920	.641	1.321
Homemade food and restaurants	1.912	**.000**	6.767	2.612	17.529
Place of residence (village)					
City	.127	.763	1.135	.497	2.593
Camp	− .126	.765	.882	.387	2.010

## Discussion

This is one of the leading studies concerning the prevalence of anxiety and depression among Palestinian university students. Most students suffer from moderate to severe depression, and around half of them had moderate to severe anxiety. This study reveals a significant increase in the prevalence of depression and anxiety among university students compared to the pre-pandemic estimates, where the severe levels of depression and anxiety were only 9.1% and 21.3%, respectively [12]. This is analogous to the results of many other studies in the USA and Greece that reported a doubling in the prevalence of anxiety and depression symptoms during the COVID-19 pandemic [13–15]. In contrast to our results, a lower prevalence of anxiety was observed among students in the United Arab Emirates [16], which could be explained by the obvious variation in the levels of citizens’ welfare and area prosperity. Also, in a Chinese study, the prevalence of anxiety and depression was lower than expected which was explained by the survey coinciding a Chinese festival [17].

The severity of anxiety was found to be significantly affected by gender, as severe anxiety symptoms were experienced by female students more than males. These results were consistent with a study conducted in China [18]. Female anxiety was related to various reasons including body image, drinking habits, and academic performance. Moreover, females had increased home responsibilities during the lockdowns [19]. The anxiety levels demonstrated a gradual increase with the student academic year which was consistent with Wang et al. [20] and Halperin et al. [21] studies. Improper sleep quality was associated with severe anxiety symptoms. These results are in concordance with a Chinese study conducted during the pandemic [18]. In 2021, Wu et al. [22] suggested that both anxiety and depression are considered to play a mediating role between sleep quality and the disorder related to eating behaviors. Smokers experienced lower percentage of severe anxiety. This could be interpreted by the fact that nicotine provides relaxing and stress—relief feeling at the short term. This led to the elevation in the smoking levels during the COVID-19 pandemic as a means to cope with the new measures and to relieve the ascending stress levels [23]. On the other hand, Zahrani et al. [24] indicated a direct relationship between smoking and anxiety levels. The source of food was found to play a role in developing anxiety. The less people dependent on homemade food, the higher they are suffering from severe anxiety. This goes with the results of a study that was performed on informal workers during the COVID-19 pandemic in Bangladesh [25]. Unhealthy food, soft drinks, and fast food were found to be associated with significant stress, depressive symptoms, and sleep disturbance [26]. In contradiction to what was expected and what was indicated by other studies like Wang et al. [20], students residing in cities were found to be the least affected by anxiety. This could be related to the fact that the Palestinian cities are relatively small compared to other large cities. Due to the special political situation, living in cities in Palestine is relatively safer than living in villages. This allows people in cities to be more comfortable and less exposed to anxiety.

Untreated anxiety might progress into depression, so it is important to study both (24). The place of residence (villages, cities, and camps) was not significantly associated with depression severity. This could be related to the similar fears that were facing the students through the COVID-19 pandemic regardless of their place of residence. This goes in line with the study of Halperin et al. [21]. The students’ academic year is a significant factor in the development of depression. Students who were in their fourth academic year or higher had shown higher tendency to develop depression than younger ones. This might be related to the fact that this stage is full of thoughts and plans for the future yet to come as students start to get themselves ready for the labor market. Higher depression prevalence is provoked by the increase in unemployment rates as a consequence of the COVID-19 pandemic [27]. Such an outcome is in harmony with a Chinese study [20]. However, a previous study in the USA has shown that students in earlier academic years had higher levels of depression [12]. These results were interpreted by the fact that those students are adapting to the novel campus life and environments as most of them relocate to live in hostels [21]. Regarding the field of study, depression levels were found to be higher among medical students than any others. This conforms with the results of Shawahneh et al. [12] which were conducted in Palestine. These results could be correlated to the fact that medical students have more academic load and stressful factors such as financial concerns and sleep deprivation [12]. Moreover, the adopted distance-learning methods during COVID-19 and its probable impact on academic performance led to increased depression among medical students. However, another study conducted in Palestine [28] had shown that depression was less prevalent among medical students than those majoring in other fields. The current study showed a direct relationship between abnormal sleeping hours and depression development which goes with an Italian study [29]. However, other studies [30] have revealed an indirect relationship between sleeping disturbance and depression. In parallel with the results of previous studies in Canada [31] and Palestine [28], the current study showed that smoking contributed to depression development. Indeed, Steuber and Danner [32] suggested that smoking leads to depression in the long term as nicotine might affect the nervous system. Students who were dependent on restaurants rather than homemade food were found to be more vulnerable to depression. These results are in line with previous studies although the specific mechanism for the improvement of psychosocial outcomes in youth who have frequent family meals are still unclear [33].

Our study revealed a strong positive correlation between GAD-7 anxiety and CES-D depression scores which is consistent with multiple previous studies [34]. The causal pathway and high rate of comorbidity were also explained by the shared genetic vulnerability, similar neurobiological features, and epiphenomenon nature between anxiety and depression [35]. These findings could be supported by the observed role of amygdala functional connectivity with other brain regions in emotion and cognitive control using functional magnetic resonance imaging and neuropsychological testing [36].

### The strengths and limitations of the study

This study has many strengths, and some limitations that should be considered when reading and interpreting the results. The strengths included the large sample size that was representative of the universities’ population size. Moreover, the sample included students from whole universities in the West Bank which provided more representative results. Furthermore, the online anonymous questionnaire is considered a strong point in studying anxiety and depression because expressing feelings of anxiety and depression is still considered a stigma which could be a barrier for the students to express their feelings if it was in person.

On the other side, some limitations were found. Conducting a web-based survey does not provide a firm diagnosis of the depression cases which makes our results of value for initial screening. Besides, this study had not excluded students with diagnosed psychiatric and physical disorders; however, the large sample size enhances the reliability and credibility of the results.

## Conclusions

The current study concluded that the prevalence of anxiety and depression among Palestinian university students is notably high. Moreover, we found a strong positive relationship between anxiety and depression. The study succeeded in determining specific variables that were significantly associated with the rise of these disorders. These results are worrying and need to be considered seriously by the stakeholders at the university as well as by the government, not only in terms of screening and diagnosing the disorders but also in terms of providing protective strategies to reduce the prevalence of these disorders. Further studies are required to determine how to maintain the students’ mental health during upcoming devastating challenges such as COVID-19 and how to relieve and support students whose mental health has been affected by the pandemic.

## Data Availability

All required data is available in this study.

## Abbreviations

CES-D: Center for Epidemiological Studies Depression
GAD-7: Generalized Anxiety Disorder scale
COVID-19: Coronavirus disease 2019
DOH: Declaration of Helsinki

## References

[1] Pandit R. Basic protective measures against the new coronavirus pandemic–COVID-19. J Manag Res Anal. 2020;7(1):1–2.

[2] Wang Z-H, Yang H-L, Yang Y-Q, Liu D, Li Z-H, Zhang X-R (2020). Corrigendum to “prevalence of anxiety and depression symptom, and the demands for psychological knowledge and interventions in college students during COVID-19 epidemic: a large cross-sectional study”[275 (2020) 188–193]. J Affect Disord.

[3] Hamdan A, Ghanim M, Mosleh R (2021). COVID-19 confinement and related well being measurement using the EQ-5D questionnaire: a survey among the Palestinian population. Int J Clin Pract.

[4] Al Zabadi H, Alhroub T, Yaseen N, Haj-Yahya M. Assessment of depression severity during coronavirus disease (2019). pandemic among the Palestinian population: a growing concern and an immediate consideration. Front Psych.

[5] Radwan A, Radwan E, Radwan W (2021). Eating habits among primary and secondary school students in the Gaza Strip, Palestine: a cross-sectional study during the COVID-19 pandemic. Appetite.

[6] Gaddis GM, Gaddis ML (1990). Introduction to biostatistics: part 2, descriptive statistics. Ann Emerg Med.

[7] Spitzer RL, Kroenke K, Williams JB, Löwe B (2006). A brief measure for assessing generalized anxiety disorder: the GAD-7. Arch Intern Med.

[8] Radloff LS (1977). The CES-D scale: a self-report depression scale for research in the general population. Appl Psychol Meas.

[9] Swinson R (2006). The GAD-7 scale was accurate for diagnosing generalised anxiety disorder. Evidence-based Med..

[10] Samreen S, Siddiqui NA, Mothana RA. Prevalence of Anxiety and Associated Factors among Pharmacy Students in Saudi Arabia: a Cross-Sectional Study. BioMed Research International. 2020;2020:2436538. 10.1155/2020/2436538. 10.1155/2020/2436538 PMC7605948 33163532

[11] Moon JR, Huh J, Song J, Kang I, Park SW, Chang S-A (2017). The center for epidemiologic studies depression scale is an adequate screening instrument for depression and anxiety disorder in adults with congential heart disease. Health Qual Life Outcomes.

[12] Shawahna R, Hattab S, Al-Shafei R, Tab’ouni M (2020). Prevalence and factors associated with depressive and anxiety symptoms among Palestinian medical students. BMC psychiatry..

[13] Racine N, McArthur BA, Cooke JE, Eirich R, Zhu J, Madigan S (2021). Global prevalence of depressive and anxiety symptoms in children and adolescents during COVID-19: a meta-analysis. JAMA Pediatr.

[14] Son C, Hegde S, Smith A, Wang X, Sasangohar F (2020). Effects of COVID-19 on college students’ mental health in the United States: interview survey study. J Med Internet Res.

[15] Kaparounaki CK, Patsali ME, Mousa D-PV, Papadopoulou EV, Papadopoulou KK, Fountoulakis KN (2020). University students’ mental health amidst the COVID-19 quarantine in Greece. Psychiatr Res..

[16] Saddik B, Hussein A, Sharif-Askari FS, Kheder W, Temsah M-H, Koutaich RA (2020). Increased levels of anxiety among medical and non-medical university students during the COVID-19 pandemic in the United Arab Emirates. Risk Manage Healthcare Policy.

[17] Wang Z-H, Yang H-L, Yang Y-Q, Liu D, Li Z-H, Zhang X-R (2020). Prevalence of anxiety and depression symptom, and the demands for psychological knowledge and interventions in college students during COVID-19 epidemic: a large cross-sectional study. J Affect Disord.

[18] Duan H, Gong M, Zhang Q, Huang X, Wan B. Research on sleep status, body mass index, anxiety and depression of college students during the post-pandemic era in Wuhan, China. J Affective Disord. 2022;301:189–92. 10.1016/j.jad.2022.01.015 PMC8721916 34990628

[19] Gao W, Ping S, Liu X (2020). Gender differences in depression, anxiety, and stress among college students: a longitudinal study from China. J Affect Disord.

[20] Wang D, Zhao J, Ross B, Ma Z, Zhang J, Fan F, et al. (2021) n2Longitudinal trajectories of depression and anxiety among adolescents during COVID-19 lockdown in China. J Affect Disord. 10.1016/j.jad.2021.12.086 PMC8691948 34952127

[21] Halperin SJ, Henderson MN, Prenner S, Grauer JN (2021). Prevalence of anxiety and depression among medical students during the Covid-19 pandemic: a cross-sectional study. J Med Educ Curric Dev.

[22] Wu R, Guo L, Rong H, Shi J, Li W, Zhu M, et al. The role of problematic smartphone uses and psychological distress in the relationship between sleep quality and disordered eating behaviors among Chinese college students. Front Psychiatry. 2021:2288. 10.3389/fpsyt.2021.793506 PMC8710586 34966312

[23] Popova L, Henderson K, Kute N, Singh-Looney M, Ashley DL, Reynolds RM, et al. (2021) “I’m bored and I’m stressed”: a qualitative study of exclusive smokers, ENDS users, and transitioning smokers or ENDS users in the time of COVID-19. Nicotine Tob Res. ntab199, 10.1093/ntr/ntab199 10.1093/ntr/ntab199 PMC8522380 34610133

[24] Alzahrani F, Alshahrani NZ, Abu Sabah A, Zarbah A, Abu Sabah S, Mamun MA. Prevalence and factors associated with mental health problems in Saudi general population during the coronavirus disease 2019 pandemic: A systematic review and meta-analysis. PsyCh J. 2022;11(1):18– 29. 10.1002/pchj.516 10.1002/pchj.516 34986503

[25] Haque MR, Khan MMA, Rahman MM, Rahman MS, Begum SA (2022). Mental health status of informal waste workers during the COVID-19 pandemic in Bangladesh. PLoS ONE.

[26] Khan A, Uddin R (2020). Is consumption of fast-food and carbonated soft drink associated with anxiety-induced sleep disturbance among adolescents? A population-based study. Clinical nutrition ESPEN.

[27] Buerhaus PI, Staiger DO, Auerbach DI, Yates MC, Donelan K (2022). Nurse employment during the first fifteen months of the COVID-19 pandemic: study examines nurse employment trends during first fifteen months of the COVID-19 pandemic. Health Aff.

[28] Safarini OA, Taya H, Elhija YA, Qadous M, Farhoud A, Thabaleh A, Khayyat A, Nazzal Z, Abuhassan AM, Ghanim N, Mahamid F. Assessment of the Relationship of Depression With Tobacco and Caffeine Use Among University Students: A Cross-Sectional Study. Cureus. 2021;13(10). 10.7759/cureus.19098 PMC8627153 34868751

[29] Viselli L, Salfi F, D’Atri A, Amicucci G, Ferrara M (2021). Sleep quality, insomnia symptoms, and depressive symptomatology among Italian university students before and during the COVID-19 lockdown. Int J Environ Res Public Health.

[30] Jiang M, Zhao Y, Wang J, Hua L, Chen Y, Yao Y, et al. Serial Multiple Mediation of the Correlation Between Internet Addiction and Depression by Social Support and Sleep Quality of College Students During the COVID-19 Epidemic. Psychiatry Invest. 2022;19(1):9. 10.30773/pi.2021.0147 PMC8795596 34986557

[31] Vieira FdST, Muraro AP, Rodrigues PRM, Sichieri R, Pereira RA, Ferreira MG (2021). Lifestyle-related behaviors and depressive symptoms in college students. Cadernos de Saúde Pública..

[32] Steuber TL, Danner F (2006). Adolescent smoking and depression: which comes first?. Addict Behav.

[33] Harrison ME, Norris ML, Obeid N, Fu M, Weinstangel H, Sampson M (2015). Systematic review of the effects of family meal frequency on psychosocial outcomes in youth. Can Fam Physician.

[34] Karadağ E, Sölpük N (2018). Relationship between depression and anxiety symptoms in studies conducted in Turkey: a meta-analysis study.

[35] Hettema JM. What is the genetic relationship between anxiety and depression? American journal of medical genetics part C: Seminars in medical genetics: Wiley Online Library; 2008. p. 140–6. 10.1002/ajmg.c.30171 18412101

[36] He C, Gong L, Yin Y, Yuan Y, Zhang H, Lv L (2019). Amygdala connectivity mediates the association between anxiety and depression in patients with major depressive disorder. Brain Imaging Behav.

